# The moderating effect of entrepreneurial motivation on the relationship between entrepreneurial intention and behaviour: An extension of the theory of planned behaviour on emerging economy

**DOI:** 10.12688/f1000research.140675.2

**Published:** 2024-07-01

**Authors:** Pushparaj M. Nayak, Harish G. Joshi, Madhukara Nayak, Mathew Thomas Gil

**Affiliations:** 1 Department of Commerce, Manipal Academy of Higher Education, Manipal, Karnataka, 576104, India; 2 Department of Mechanical Engineering, Shri Madhwa Vadriraja Institute of Technology and Management, Bantakal, India, karnataka, 574115, India

**Keywords:** Entrepreneurial intentions; entrepreneurial behaviour, theory of planned behaviour, higher education institution; structural equation modelling, engineering students.

## Abstract

**Background:**

The study investigates the influence of antecedents of the theory of planned behaviour (TPB) and personality traits on entrepreneurial intention and behaviour among engineering students in an emerging economy. It employs the extension of the TPB model by focusing on the intention-behaviour gap, an under-researched area in research. Furthermore, it investigates the moderating effect of entrepreneurial motivation on the relationship between intention and behaviour to improve conceptual model predictability.

**Methods:**

A structured questionnaire was used to gather data from 1,564 engineering students, and the data were analyzed using structural equation modelling (SEM) with Amos software.

**Results:**

The results revealed that subjective norms were the strongest predictor of entrepreneurial intention and behaviour, followed by entrepreneurial alertness, perceived behavioural control, attitude towards entrepreneurship, need for achievement, and risk tolerance. Moreover, the moderation analysis showed that entrepreneurial motivation was crucial in moderating the relationship between intention and behaviour.

**Conclusions:**

The present conceptual model contributes to the existing TPB model by improving predictive power to understand the intention-behaviour relationship. The results of the study will assist policymakers, academicians of higher education institutions, and universities in developing policies, strategies, and curricula to engage more students in entrepreneurial activities.

## Introduction

Entrepreneurship has become widely popular because of its ability to promote economic activity and contribute to the overall economic development of a region or country ( Veleva, 2020). Entrepreneurs are viewed as individuals who foster economic growth by generating and implementing innovative ideas into successful business enterprises ( Turker and SonmezSelcuk, 2009, Voutsina, Papagiannakis and Lioukas, 2022). The growth of entrepreneurship is crucial for generating job opportunities, promoting innovation, and improving the overall efficiency of various economic sectors (Gonçalo Rodrigues Brás, Daniel and Fernandes, 2023; Robert, Frey and Sisodia, 2021). According to the Global Entrepreneurship Monitor (GEM) 2021–2022 survey report, the world's largest research organization on entrepreneurship, there was a significant increase in total entrepreneurial activity (TEA) in India from 5.30% in 2020 to 14.40% in 2021. In addition, India has made remarkable progress in the “ease of doing business” parameter, achieving a fourth rank in 2021, up from its fifth rank in 2020. According to the Times of India 2020 report, 83% of the Indian workforce (aged 25–34) expressed a desire to begin their own businesses, notably higher than the global average of 53%. According to Mukesh, Rao and Rajasekharan Pillai K. (2018), there is a significant disconnect between the entrepreneurial potential of students in India and the higher education system. However, Pandit, Joshi and Tiwari (2018) suggested that entrepreneurship education has the potential to cultivate student interest and commitment towards entrepreneurship as a career path. Thus, nurturing the entrepreneurial intention (EI) of young individuals has become an essential requirement for fostering the growth and development of entrepreneurship in any country.

Krueger and Carsrud (1993) defined EI as the level of determination to engage in the necessary behaviors required to physically initiate a business venture. Baum and Locke (2004) emphasized that entrepreneurship is an intentional process, which is a fundamental aspect of entrepreneurship research. Yar Hamidi, Wennberg and Berglund (2008) conducted empirical studies and concluded that an individual's intention to pursue entrepreneurship is a powerful indicator of his or her future participation in entrepreneurial activities. A few predominant theories in entrepreneurial decision making are ( Bandura, 1977) Social Cognitive Theory (SCT) ( Tversky and Kahneman, 1974) Dual dual-process theory, Shapero and Sokol (1982), Entrepreneurial Event Theory (EET), and Ajzen’s (1991) theory of planned behavior (TPB).

The TPB is a highly influential and frequently employed theoretical model for examining human actions ( Ajzen, 2020). Additionally, TPB has been widely employed as a theoretical framework for investigating the EI of students in multiple contexts and cultures ( Hosen *et al*., 2022; Kumar and Shukla, 2023; Zellweger, Sieger and Halter, 2011), and was selected as the research framework for this study. According to Ajzen (1991), Kolvereid (1996), and Krueger, Reilly and Carsrud (2000), the TPB model highlights three motivational factors, commonly referred to as antecedents that impact the formation of intentions to engage in behaviors: attitudes towards behavior (ATB), subjective norms (SN), and perceived behavioral control (PBC). Numerous studies have indicated that ATB, SN, and PBC typically account for 30-50% of the variance in intention, implying that approximately half of the variance in EI remains unexplained ( Kolvereid, 1996; Krueger, Reilly and Carsrud, 2000; Liñán and Chen, 2009). Liñán *et al*. (2010) and other scholars have suggested that incorporating additional variables could help close the gap in the unexplained variance. Therefore, researchers have incorporated additional variables, such as need for achievement (NACH), risk tolerance (RT), and entrepreneurial alertness (EA), into the original TPB model to address its limited explanatory power. NACH, RT, and EA are personality characteristics that can drive an individual to develop the intention to become an entrepreneur. Earlier studies conducted in various countries have established a connection between personality traits such as NACH, RT, and EA and EI ( Li *et al*., 2020; Mahmoodi *et al*., 2023; Marques and Fuinhas, 2012). However, only a limited number of empirical investigations have explored the impact of these factors on students' EI ( Ouni and Boujelbene, 2023; Passah and Panda, 2021). Few studies have examined the combined impact of personality traits and TPM on students' EI, especially in the Indian context. Thus, the first objective of this study is to examine the influence of personality traits and antecedents of TPB on the EI and behavior of engineering students in India.

Many studies on entrepreneurship have used intentions as the dependent variable; however, they have not adequately examined the relationship between intentions and behavior ( Gull *et al*., 2021). Moreover, evidence suggests that not all business intentions are transformed into actual behavior or new venture creation. Although research in other fields has found a positive correlation between intention and behavior, there is a lack of evidence on the intention-behavior linkage, specifically in the context of entrepreneurship ( Thomas, 2022). Therefore, the main challenge of entrepreneurship research is to fill the knowledge gap in the intention-behavior linkage ( Gieure, Benavides-Espinosa and Roig-Dobón, 2020). Although a link between intention behavior and motivation may exist, the same has not been tested in terms of the extension of the TPB ( Carsrud and Brännback, 2010). Researchers have recommended a longitudinal study to examine the transformation of intention into behavior ( Farooq *et al*., 2018). However, applying a longitudinal study to engineering students presents challenges, as an engineer's role in the industry evolves with experience and career advancement. Initially, engineers tended to have more technical roles, which transformed into managerial positions over time. Therefore, a longitudinal study may not be an immediate solution for investigating the transformation of intention into behavior. Hence, the second objective of this study was to investigate how entrepreneurial motivation moderates the link between intention and behavior. In this way, our findings are expected to contribute to the scholarly debate on the linkage between EI and behavior. The study proposes practical suggestions for policymakers and academicians on how to advance training content and design the curricula as a result of this research.

This study had two main goals. First, it examines how antecedents of planned behavior (TPB) and personality traits influence entrepreneurial intention and behavior among engineering students in an emerging economy. Second, this study investigates the moderating effects of entrepreneurial motivation on the relationship between entrepreneurial intention and behavior. The paper begins with a literature review that covers earlier research, followed by hypotheses and the proposed model for the study. The research methodology is then presented to outline the scope of the study, followed by data analysis. The last section includes the conclusion, which discusses the results, implications, further research opportunities, and limitations of the study.

## Literature review

### Attitude towards entrepreneurship (ATE) and entrepreneurial intention (EI)

According to Ajzen (1991) theory, a person's attitude, whether affective/experiential (feelings of joy or satisfaction) or instrumental/cognitive (beliefs, thoughts, or rational arguments), is a key determinant in initiating any action. Ajzen (1991, 2002) and Kolvereid (1996) suggested that an individual's ATE reflects their personal evaluation of desirability, indicating the degree to which they hold a positive or negative view of being an entrepreneur. An individual's attitude is a predisposition that influences their positive or negative responses towards an object, person, institution, or event. This attitude plays a significant role in shaping an individual's behavioral intentions ( Ajzen and Driver, 1992; Bell and Cui, 2023; Thoudam *et al*., 2021). The assessment of an individual's ATE indicates their perception of the positive or negative consequences associated with engaging in entrepreneurial activities ( Esfandiar *et al*., 2019). When considering different career options, an individual considers various potential consequences such as financial gain, risk factors, and level of autonomy ( Douglas and Shepherd, 2002; Gibson *et al*., 2021). According to Lumpkin and Dess (1996), entrepreneurship involves various types of risks, such as personal, social, and psychological, while independence pertains to the level of autonomy in decision-making, and income encompasses both material and non-material benefits of entrepreneurship. Although some studies ( Gultom *et al*., 2020; Zahid and Haji Din, 2019; Zainuddin and Mukhtar, 2022) have examined the relationship between ATE and EI, we aim to test this relationship in the Indian context to confirm its validity, as previous research has not extensively examined this relationship in India. Thus, we propose the following hypothesis:

**H1**: ATE significantly influences EI.

### Subjective norm (SN) and entrepreneurial intention (EI)

Subjective norm (SN), a construct associated with TPB, is believed to predict intentions as it comprises an individual's beliefs, such as whether others think they should perform a particular behavior ( Conner and Armitage, 1998). Regarding entrepreneurship, SN measures the perceived social pressure an individual feels to become an entrepreneur, which can come from significant people, such as family members, friends, teachers, and financiers. The strength of an individual's motivation to comply with these perceptions also plays a role ( Krueger *et al*., 2000; Yasir *et al*., 2023).

There needs to be more consensus among scholars in the literature on the impact of SN on EI. While some studies have suggested that SN has a weaker influence than ATE and PBC ( Autio *et al*., 2001; Echchabi, Ayedh and Omar, 2020; Ruizalba Robledo *et al*., 2015), other studies have demonstrated a significant relationship between SN and EI ( Gultom *et al*., 2020; Khan *et al*., 2020; Kolvereid, 1996; Ruiz-Dotras and Lladós-Masllorens, 2022). According to Hofstede (2011), the results regarding the relationship between SN and EI can be divided into socialistic and individualistic countries. This study found a positive correlation between SN and EI in socialistis countries such as Russia, Ghana, and Southeast Asian countries. However, in the context of the USA and the EU, a negative correlation or no significant correlation was observed between SN and EI. Considering the communal lifestyle, the limited job opportunities available in the public sector, and the perceived difficulties associated with entrepreneurship in an industrially underdeveloped region with corrupt practices related to setting up a business ( Panda, 2000), investigators are encouraged to reconsider the correlation between SN and EI in the Indian context. Dubey and Sahu (2022) found that the collectivistic orientation of Indian culture positively influences entrepreneurial intention through its effect on subjective norms and perceived behavioral control. The authors argue that in a collectivist society, the approval and support of family and friends hold greater importance in shaping an individual's entrepreneurial aspirations. Similarly, Gupta and Bhawe (2007) explored the influence of cultural dimensions such as uncertainty avoidance and power distance on entrepreneurial intentions among Indian engineering students. Their findings suggest that lower levels of uncertainty avoidance and power distance are associated with higher entrepreneurial intention. This indicates that cultural traits that promote risk-taking and autonomy can foster entrepreneurial intention in the Indian context. Drawing on the literature review results, Hypothesis H2 was formulated in the following manner.

**H2:** SN significantly influences EI.

### Perceived behavioural control (PBC) and entrepreneurial intention (EI)

According to Boyd and Vozikis (1994), PBC, the third construct of the TPB, plays a critical role in assessing the magnitude of both EI and entrepreneurial behaviors, according to Boyd and Vozikis (1994) research. Ajzen (1991) defined PBC as an individual's perception of the level of difficulty or ease in performing behavior, taking into account anticipated obstacles and challenges, as well as previous experiences. According to Ajzen (2002), PBC is a more comprehensive construct than self-efficacy, as it considers not only internal factors, such as knowledge, skills, and confidence, but also external factors, such as resources, opportunities, and potential barriers. PBC predicts behavior directly, whereas self-efficacy only predicts intentions ( Armitage and Conner, 2001). Boyd and Vozikis (1994) suggested that self-efficacy, as a control belief, serves as the foundation for an individual's perception of behavioral control. This perception is based on preconceived notions of the availability or unavailability of necessary resources and opportunities ( Ajzen, 2001). In addition to differences in conceptualization and operationalization, the construct has become controversial because of the lack of consistent empirical evidence supporting its impact on intention ( Yap, Othman and Wee, 2013). Although the positive impact of PBC on EI has been extensively studied in other countries (Giovanni Di Stefano *et al*., 2023; Krueger and Carsrud, 1993; Ruizalba Robledo *et al*., 2015), the present study aims to revisit its impact in the specific context of India, where factors such as perceived government support, family support, entrepreneurship development programs, and service quality of entrepreneurship education may affect EI with varying degrees of influence. Drawing on the aforementioned considerations, we formulated Hypothesis H3 in the following manner.

**H3**: PBC significantly influences EI.

### Entrepreneurial alertness (EA) and entrepreneurial intention (EI)

EA is an essential trait of entrepreneurs. It refers to the ability of individuals to identify and recognize entrepreneurial opportunities. Several previous studies have reported a positive relationship between EA and EI ( Tang *et al*., 2012; Hu *et al*., 2018; Neneh, 2019). EA has garnered significant attention in entrepreneurship as it facilitates the identification of suitable career paths and enables the exploitation of entrepreneurial opportunities ( Tang, 2008; Short *et al*., 2009; Xin and Ma, 2023). According to Montiel Campos (2017), EA begins with an individual's capability to identify potential opportunities, followed by the continuous personal growth necessary to transform those opportunities into actual outcomes. McMullen and Shepherd (2006) suggested that EA should lead to entrepreneurial action. Lu and Wang (2018) identified a direct relationship between EA and EI. This is because EA helps individuals recognize business opportunities and make informed judgments, which, in turn, influences their intent to pursue entrepreneurship. In addition, several scholars have pointed out that alertness is an essential skill for entrepreneurs to anticipate and recognize opportunities ( Ardichvili and Cardozo, 2000; Mannino and Schiera, 2017; Shamsudeen, Keat and Hassan, 2017). Thus, it can be inferred that individuals who possess a higher level of alertness are more likely to identify favorable opportunities and embark on entrepreneurial careers. Based on this observation, this study proposes the following hypothesis:

**H4**: EA significantly influences EI.

### Need for achievement (NACH) and entrepreneurial intention (EI)

McClelland (1961) proposed the need for achievement, a personality trait characterized by a strong aspiration to establish and sustain elevated performance levels. People with a high need for achievement are highly motivated to succeed, set higher goals, take calculated risks, and select innovative and moderately tricky tasks that are challenging, yet achievable. Entrepreneurial careers allow for greater control over outcomes, entail moderate risk taking, and offer immediate feedback on performance. It is logical to anticipate that individuals with a strong need for achievement will be inclined to pursue entrepreneurship as a profession. McClelland (1965) conducted a longitudinal study that revealed that a greater number of individuals with a high need for achievement scores were engaged in entrepreneurial occupations compared to those with lower scores on the same trait. Mukesh, Pillai and Mamman (2019) found that NACH is a significant predictor of EI among engineering students in India. Other studies, such as Chaudhary (2017) and Littunen (2000), also reported that NACH has a stronger link with entrepreneurship than any other trait.

In contrast, Davidsson and Wiklund (1999) argued that the need for achievement traits has limited significance in predicting entrepreneurial behavior. These conflicting results highlight the necessity for further investigation of the relationship between the need for achievement traits and entrepreneurial intention. Consequently, we propose the following hypothesis:

**H5:** NACH significantly influences EI.

### Risk Tolerance (RT) and Entrepreneurial Intention

Risk-taking propensity is often considered an important trait for entrepreneurs as it involves the willingness to take risks in the face of uncertainty. Entrepreneurs face many uncertain situations and must make decisions without complete information; a high tolerance for risk can help them navigate these challenges. Brockhaus (1980) was one of the first to suggest that the risk-taking propensity is an important trait for entrepreneurs. Since then, many studies have reported a positive relationship between risk-taking propensity and entrepreneurial intention or behavior ( Liñán and Chen, 2009; Rauch and Frese, 2007). However, it's important to note that not all entrepreneurs are high-risk takers, and there is some debate over the extent to which risk-taking propensity is a necessary trait for entrepreneurship ( Busenitz and Lau, 1996; Mitchell *et al*., 2002; Sobaih and Elshaer, 2023). Therefore, we hypothesize the following:

**H6:** RT significantly influences EI.

### Moderating role of entrepreneurial motivation (EM) on the relationship between entrepreneurial intention (EI) and entrepreneurial behaviour (EB)

There are significant differences between the intentions and behaviors of real start-ups. Although scholars advise longitudinal studies to assess the ratio of entrepreneurial intention to actual behavior ( Farooq *et al*., 2018), the difference cannot be determined using conventional research methods. The objective of this study is to transform intentions into actions. In the 1980s, Sexton, Donald and Smilor, Raymond (1986) and Smilor and Kuhn (1986) conducted preliminary theoretical and practical viewpoints. Subsequently, research on entrepreneurs' characteristics took the lead, and EM research could no longer attract scholars.

Bird (1989) and Krueger and Carsrud (1993) stressed the importance of transforming intention into behavior in realizing the complete process of entrepreneurship. The attitude-intention and intention behavior links define the relationship between attitude and behavior. Scholars have not focused on empirical studies on the relationship between entrepreneurial motivation and behavior ( Kuratko *et al*., 2017), but a previous study by Carsrud, Olm and Thomas (1989) addressed motivation and behavior in the context of business performance. According to Carsrud and Brännback (2010) EM creates a relationship between intention and action. Motivators are impulses that ultimately drive actions in pursuit of a goal. Carsrud and Brännback (2010) further found that scholars have researched motivation to explain different reactions of people to the same stimuli of motivation and choice of diverse personal behavior.

Push and pull motivation theories are two types of motivation theories. These pull factors motivate individuals to engage in entrepreneurial endeavors ( CantúCavada, Bobek and Maček, 2017; Iqbal *et al*., 2020). Pull factors include the ability to employ others, social status, the opportunity to use one's education and experience, the support and encouragement of one's family, independence, the potential to learn new skills, market opportunities, financial independence, more negotiating power at home, and more control over household decisions ( Chhabra, Raghunathan and Rao, 2020; Lockyer and George, 2012). Push factors motivate individuals to engage in entrepreneurial activities ( Zgheib, 2018). Push factors include lack of job satisfaction, low household earnings, insufficient pay, and necessity ( Dawson and Henley, 2012).

This goal is essential for motivational studies ( Lockyer and George, 2012). Goals are intangible, representing future results and motivating people to continue working hard ( Chhabra, Raghunathan and Rao, 2020). Motivation also acts as a link between intention and behavior. The capacity of individuals to adapt to changing environments originates from their ability to modify their motivation and goals ( Chaudhary, 2017).

According to Ryan and Deci (2000), motivation is influenced by a combination of an individual's cognitive processes, and natural and social factors. Motivational initiatives set the direction fo purpose and determination. Therefore, the pursuit of motivation, which is driven by an individual's goals and motives, serves as a crucial factor in bridging the gap between intention and behavior. Earlier research studies suggest that there is a considerable time lag between the formation of intentions in individuals and their actual manifestation in behavior ( Helmreich *et al*., 1986). Ajzen (1991) posited that an individual's behavioral intention is influenced by their ATB, SN, and PBC, which, in turn, leads to the actual manifestation of the behavior. A link between intention behavior and motivation may exist, but the same has not been tested in terms of the extension of the TPB ( Carsrud and Brännback, 2010). Scholars have highlighted the lack of research on this particular aspect of entrepreneurship, which has been addressed in the present study, which aims to elucidate engineering students' intentions, motivations, and behaviors. As a result, the following hypothesis was developed:

**H7:** EM moderates the relationship between EI and EB.

**Figure 1.**
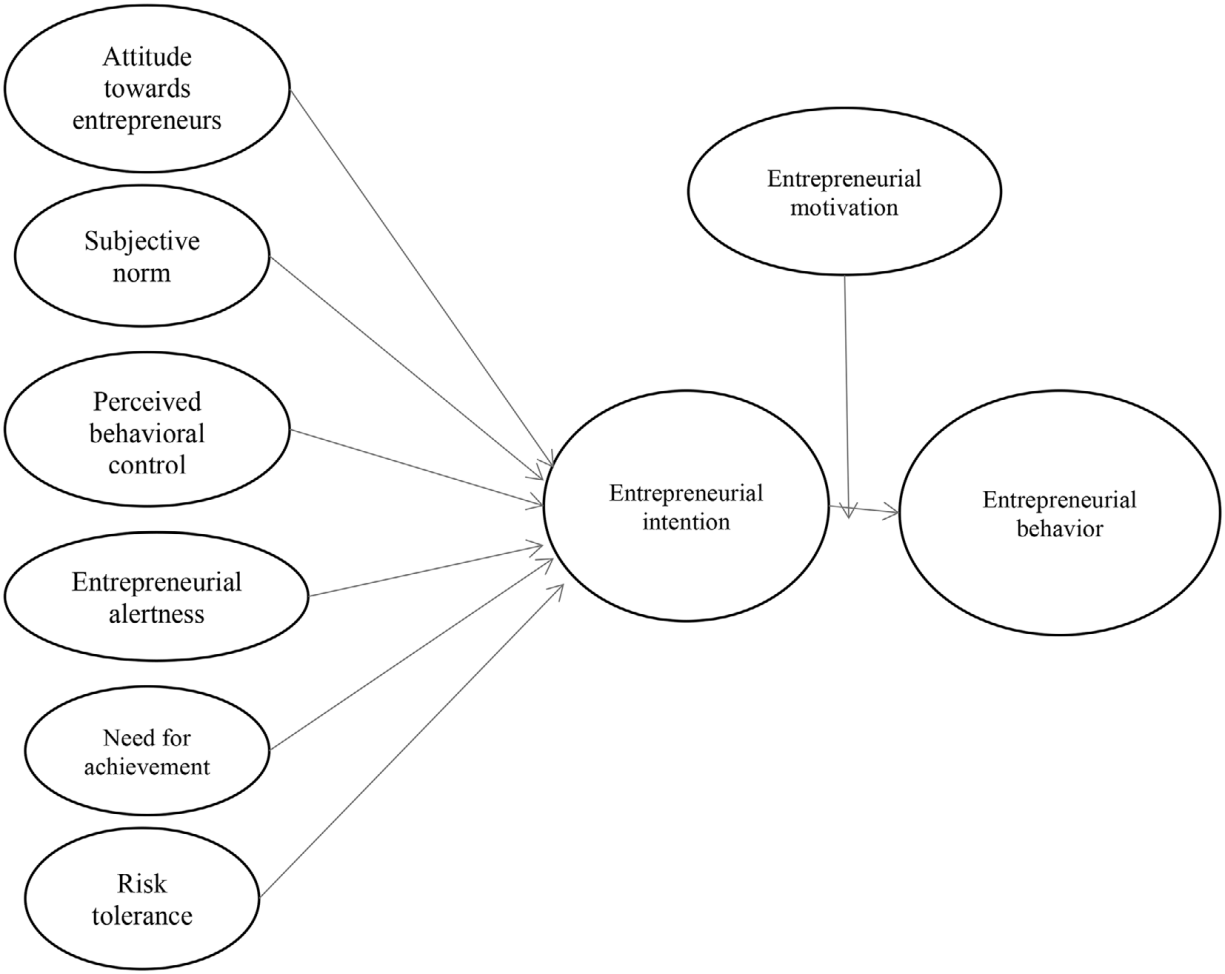
Conceptual framework for the study.

Figure 1 illustrates the conceptual framework developed based on the hypotheses discussed above.

## Methods

### Common method bias (CMB)

The CMB needs to be assessed when both independent and dependent variables are assessed using the same survey instrument. The CMB was assessed using Harman’s single-factor analysis ( Lee *et al*., 2014). The results of this test showed that a single factor explained 34.99% of the total variance. Because this value is significantly less than 50%, it is safe to assume that there is no dominant factor in the dataset. Hence, it is proven that the CMB issue does not exist in the collected samples.

### Research design

A survey questionnaire was used as the research instrument (see *Extended data;* Nayak, 2023b). All the items used to measure the constructs were adopted from previous studies. Attitudes towards entrepreneurship, subjective norms, and entrepreneurial intention were measured using a 5-item, 3-item, and 4-item scale adapted from Liñán *et al*. (2010). Perceived behavioral control and risk tolerance were measured using a 5-item and 7-item scale adapted from Chatterjee, Das and Srivastava (2019). Need for achievement was measured using an 8-item scale developed and adapted by Chatterjee, Das and Srivastava (2019) and Dinis *et al*. (2013). Entrepreneurial alertness was measured using the 4-item scale developed and adopted by Tang *et al*. (2012). Entrepreneurial motivation was measured using a 4-item scale developed and adapted from Barba-Sánchez and Atienza-Sahuquillo (2012). Entrepreneurial behavior was measured using a 4-item scale developed and adopted by Li *et al*. (2020). All items were measured on a 5-point Likert scale, with 1 indicating “strongly disagree” and 5 indicating “strongly agree.”

### Sampling design

A quantitative approach was applied to accomplish the research objectives and test the proposed research model. A cross-sectional descriptive research design was used in the study. Data were collected from final-year engineering students studying in several colleges across India using a structured questionnaire adapted from earlier studies. The questionnaires were personally distributed to the students, who were informed that participation in the survey was voluntary. They were also assured that their responses would be used only for academic purposes and were kept confidential. This study distributed 2000 hard copies of the survey questionnaire, and 1564 usable questionnaires were further processed for data analysis, yielding a response rate of 78.2%.

### Statistical analysis

The proposed research model was analyzed using SEM supported by the AMOS and SPSS software. According to Hair *et al*. (2010), the analysis of moments structure model (AMOS) is one of the latest software packages. Developed and available in the market, this software is used to assist researchers in performing analysis of inter-relationships and to create models for such inter-relationships within constructs that possess multiple indicators in an efficient, accurate, and effective manner. A confirmatory factor analysis was used to evaluate the reliability and validity of each construct in the model. The criteria to consider the model achieve overall fit with actual data when CFI, GFI, TLI, and IFI are all greater than 0.9 and RMSEA is less than 0.08 ( Hu and Bentler, 1999). The factor loadings of the items within each construct were greater than 0.5, indicating that the constructs in the model achieved convergent validity. The constructs achieve reliability when the composite reliability (CR) and Cronbach's alpha are greater than 0.6, and the average variance extracted (AVE) is greater than 50% ( Fornell and Larcker 1981; Lee *et al*., 2013). To test discriminant validity between constructs in the model, we compared the square root value of AVE and correlation coefficients in the model or used a 95% confidence interval of correlation coefficients ( Fornell and Larcker, 1981). If the square root of the AVE values of each construct is greater than the correlation of constructs, or the 95% confidence interval of the correlation coefficient does not contain one value, it indicates that the constructs have discriminant validity. We used structural equation modeling to test the hypotheses with statistically significant criteria at a level of 5%.

## Results

According to Hair *et al*. (2010), SEM can be effectively evaluated using a two-step approach that first assesses the measurement model and then examines the proposed structural model.

### Assessment of measurement model

We used confirmatory factor analysis (CFA) to test the properties of our measures using a saturated model (final model). The results showed that the model achieved an overall fit with the actual data: CFI = 0.958; GFI = 0.949; TLI = 0.929; IFI = 0.947; all were larger than 0.9, and RMSEA = 0.041 was less than 0.08 ( Nayak, 2023a).

All constructs had factor loadings higher than the benchmark level of 0.05, which indicated that the constructs achieved convergent validity. The Cronbach's alpha and composite reliability coefficients of all constructs exceed the 0.7 benchmarks, and all AVEs were larger than 0.5 ( Table 1). These tests demonstrated that our constructs achieved internal consistency and reliability.

**Table 1.** Measurement Model Analysis.

Constructs	Items	Construct Loading	Cronbach’s Alpha	CR	AVE
Attitude Towards Entrepreneurship (ATE)	ATE1	.718	0.902	0.884	0.604
	ATE2	.808			
	ATE3	.759			
	ATE4	.869			
	ATE5	.722			
Subjective Norm (SN)	SN1	.812	0.866	0.873	0.696
	SN2	.840			
	SN3	.850			
Perceived Behavioral Control (PBC)	PBC1	.823	0.912	0.903	0.650
	PBC2	.779			
	PBC3	.829			
	PBC4	.805			
	PBC5	.794			
Need for Achievement (NACH)	NACH1	.777	0.896	0.927	0.613
	NACH2	.803			
	NACH3	.823			
	NACH4	.829			
	NACH5	.724			
	NACH6	.701			
	NACH7	.795			
	NACH8	.804			
Risk Tolerance (RT)	RT1	.649	0.888	0.860	0.507
	RT2	.667			
	RT3	.719			
	RT4	.692			
	RT5	.742			
	RT6	.745			
	RT7	.702			
Entrepreneurial Alertness (EA)	EA1	.694	0.878	0.870	0.627
	EA2	.804			
	EA3	.816			
	EA4	.845			
Entrepreneurial Intention (EI)	EI1	.783	0.893	0.899	0.690
	EI2	.838			
	EI3	.866			
	EI4	.834			
Entrepreneurial Behavior (EB)	EB1	.880	0.867	0.906	0.708
	EB2	.747			
	EB3	.865			
	EB4	.867			
Entrepreneurial Motivation (EM)	EM1	.749	0.913	0.919	0.655
	EM2	.840			
	EM3	.822			
	EM4	.786			
	EM5	.859			
	EM6	.795			

*Note:* CR = composite reliability; AVE = average variance extracted.

**Figure 2.**
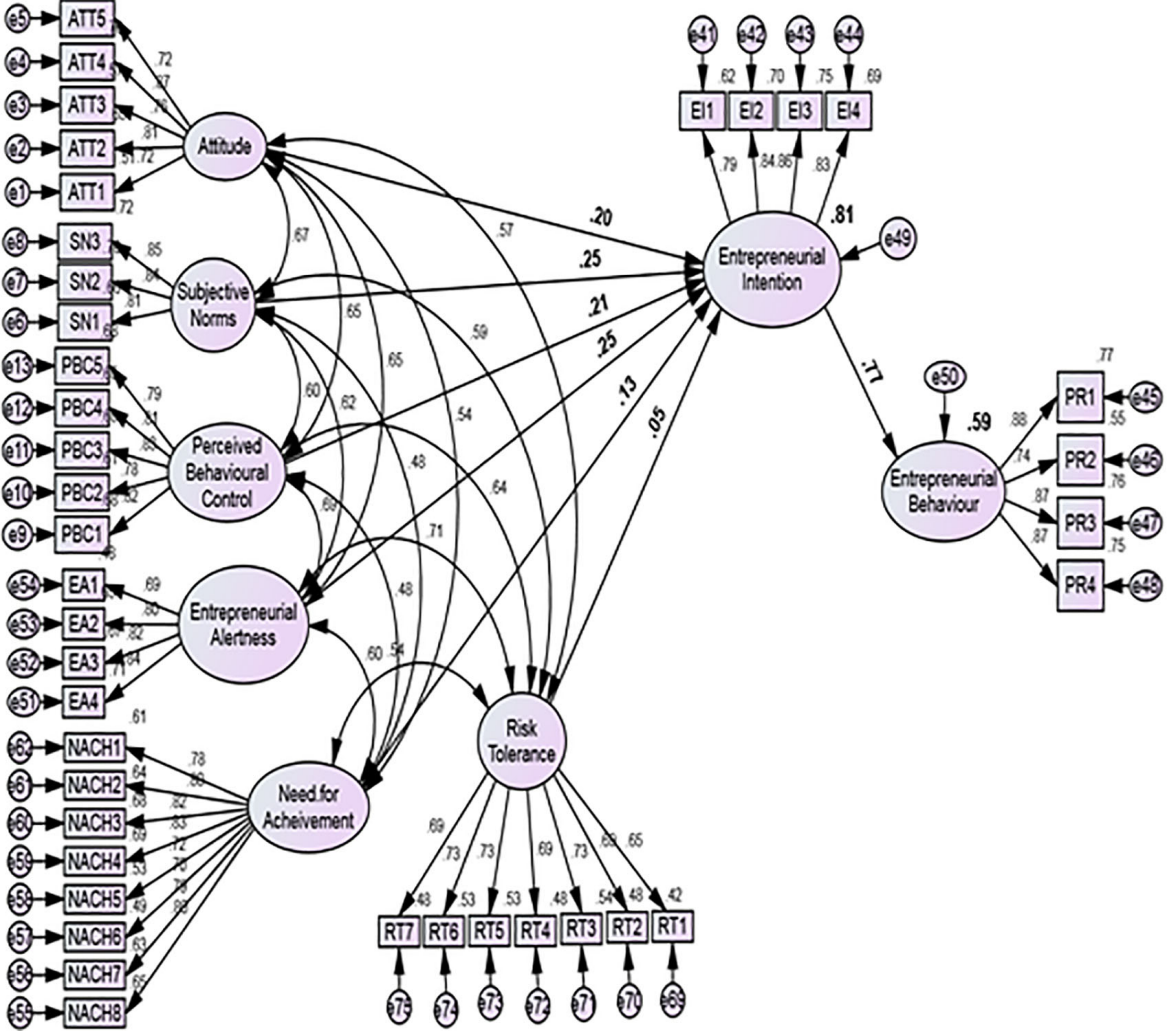
Structural model analysis.

The analysis result indicated that all constructs have the square root of AVE values of all the constructs was greater than the inter-construct correlations ( Table 2 and Figure 2). Therefore, based on the Fornell–Larcker criterion, it was proven that adequate levels of discriminant validity exist in the measurement model.

**Table 2.** Discriminant Validity (Fornell–Larcker Criterion).

	EB	ATE	SN	PBC	NACH	EA	RT	EI	EM
EB	**0.796**								
ATE	0.308	**0.777**							
SN	0.431	0.665	**0.834**						
PBC	0.468	0.654	0.599	**0.806**					
NACH	0.219	0.541	0.475	0.483	**0.783**				
EA	0.491	0.651	0.624	0.690	0.603	**0.792**			
RT	0.408	0.574	0.585	0.636	0.551	0.711	**0.712**		
EI	0.485	0.774	0.744	0.745	0.651	0.782	0.691	**0.831**	
EM	0.451	0.461	0.460	0.564	0.449	0.436	0.551	0.445	**0.809**

*Note:* Bold diagonals indicate square root of AVE. EB = entrepreneurial behaviour; ATE = attitude towards entrepreneurship; SN = subjective norms; PBC = perceived behavioural control; NACH= need for achievement; EA = entrepreneurial alertness; RT = risk tolerance; EI = entrepreneurial intention; EM= entrepreneurial motivation; AVE = average variance extracted.

### Assessment of structural model

After successfully validating the measurement model, researchers proceeded to the next stage of their analysis to evaluate the structural model and test their hypotheses about the relationships between latent variables. To assess the appropriateness of the proposed model, the researchers examined the R-squared value of the structural model. The results showed that the constructs ATE, SN, PBC, NACH, RT, and EA explained 81% of the variance in EI. Additionally, the impact of EI on behavior was measured and found to be 59%.

Table 3 shows the results of the hypothesis testing. The results showed that all the constructs were significant predictors of EI. Furthermore, SN (Path Coefficients = 0.252, CR = 9.993, P = 0.000) and EA (Path Coefficients = 0.246, CR = 8.044, P = 0.000) showed a relatively strong effect on EI, followed by PBC (Path Coefficients = 0.208, CR = 8.020, P = 0.000) and ATE (Path Coefficients = 0.198, CR = 7.482, P = 0.000). However, NACH (Path Coefficients = 0.127, CR = 6.203, P = 0.000) and RT (Path Coefficients = 0.054, CR = 2.073, P = 0.038) showed the lowest influence on EI.

**Table 3.** SEM results.

Hypothesis Statement			Estimate	CR	P value	Significance
EI	←	ATE	0.198	7.482	0.000	Supported at p<0.01
EI	←	SN	0.252	9.993	0.000	Supported at p<0.01
EI	←	PBC	0.208	8.020	0.000	Supported at p<0.01
EI	←	NACH	0.127	6.203	0.000	Supported at p<0.01
EI	←	RT	0.054	2.074	0.038	Supported at p<0.05
EI	←	EA	0.246	8.044	0.000	Supported at p<0.01
EB	←	EI	0.769	29.253	0.000	Supported at p<0.01

*Note:* ATE = attitude towards entrepreneurship; SN = subjective norms; PBC = perceived behavioral control; NACH = need for achievement; RT = risk tolerance; EA = entrepreneurial alertness; EI = entrepreneurial intention; EB = entrepreneurial behavior.

### Moderating effect of entrepreneurial motivation (EM)

According to Table 4, the direct influence of entrepreneurial motivation (EM) on entrepreneurial behaviour (EB) is not statistically significant, indicating that EM alone may not significantly impact EB. Nevertheless, the study revealed that the interaction between entrepreneurial intention (EI) and EM (EI x EM) is significant, with a p-value less than 0.05. This outcome supports the hypothesis that the relationship between EI and EB is moderated by EM, implying that the influence of EI on EB is reliant on the level of EM. To further examine this relationship, the researchers created a plot illustrating the three variables at three levels of EI and EM based on their means and standard deviations. These levels include low, medium, and high, providing a better understanding of how the interaction between EI and EM affects EB at varying levels.

**Table 4.** Moderating effect of entrepreneurial motivation on entrepreneurial behaviour.

Dependent variable	Independent variable	Regression coefficient	Standard error	t-statistic (p-value)	R square
EB	Constant	0.413	0.243	1.697 (0.089)	0.531
	EI	0.3663	0.078	4.654 (0.000)	
	EM	-0.0439	0.0718	-0.610 (0.541)	
	Interaction variable	0.1059	0.0208	5.0932 (0.000)	

*Note:* EM = entrepreneurial motivation; EI = entrepreneurial intention; EB = entrepreneurial behaviour.

**Figure 3.**
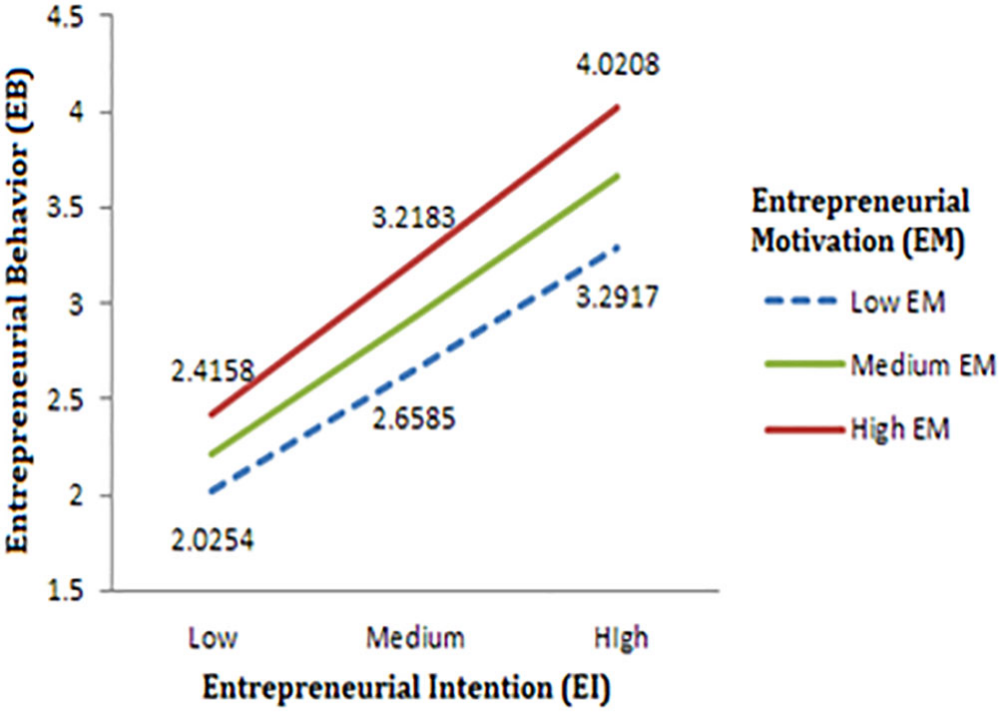
Moderating effect of EM on the relation between EI and EB.

The results obtained from simple slope analyses, as shown in Figure 3, suggest that there is a positive relationship between entrepreneurial intention (EI) and entrepreneurial behavior (EB) among the engineering students who participated in the study. The plot further reveals that higher levels of entrepreneurial motivation (EM) correspond to higher EB values for a given EI level, indicating that motivation amplifies the impact of EI on EB. Moreover, as the level of EI increased, the difference between the plots for various levels of EM also increased, implying that the effect of motivation on EB was more pronounced at higher levels of EI. Thus, the findings suggest that students with high levels of EI and EM are more likely to exhibit entrepreneurial behavior.

## Discussion, implication and conclusion

While prior research has extensively investigated the impact of antecedents of the theory of planned behavior (TPB) on entrepreneurial intention (EI) among higher education students in various contexts ( Hassan *et al*., 2021; Hoang *et al*., 2020), there is still a gap in understanding the combined effect of personality traits and TPB antecedents on EI among engineering students in South Asian emerging economies. This is particularly significant, as entrepreneurship development can vary across different regions of the world. As such, this study aimed to explore the influence of personality traits and TPB antecedents on engineering students' EI and behavior. The study also examined the moderating effect of entrepreneurial motivation on the relationship between intention and behavior.

The study found that subjective norm (SN) (β = 0.252) was the biggest determinant of EI among engineering students in India, which contrasts with previous research conducted in individualistic societies ( Bazkiaei *et al*., 2021; Boutaky and Sahib Eddine, 2022; Krueger, Reilly and Carsrud, 2000; Liñán and Chen, 2009). Earlier studies have shown that SN is a weak predictor of EI. However, the study suggests that in collectivistic cultures like India, students may be more susceptible to external influences such as peer pressure, societal expectations, and guidance from relatives and teachers when it comes to pursuing entrepreneurship ( Shrivastava and Acharya, 2020). This is because Indian culture places a significant emphasis on family, friends, and society in shaping an individual's beliefs and behaviors ( Marmat, 2021). Another notable study by Hsu *et al.* (2019) examined the role of entrepreneurial passion in shaping entrepreneurial intention among a sample of engineering students in Taiwan. The results indicate that entrepreneurial passion, along with entrepreneurial self-efficacy and perceived feasibility, positively influences entrepreneurial intention. This study highlights the importance of considering emotional and motivational factors to understand the antecedents of entrepreneurial intention, indicating that interventions aimed at encouraging entrepreneurship among engineering students in India should focus on addressing the social barriers and norms that discourage entrepreneurship and promote a positive attitude towards entrepreneurship among family members and peers. These findings have significant implications for entrepreneurship education and policymaking as they suggest the importance of creating supportive environments that encourage and facilitate entrepreneurial activity among students.

The study found that entrepreneurial alertness (EA) (β = 0.246) was the second most important factor affecting students’ EI, in line with previous research ( Lim, Lee and Mamun, 2021; Minola, Criaco and Obschonka, 2015; Neneh, 2019). This suggests that individuals who are more alert to entrepreneurial opportunities and are better equipped to recognize and act on them are more likely to form EI and behaviors. By improving an individual's ability to search and scan, gather the right information, and identify opportunities, EA can increase the likelihood of starting their own businesses and becoming entrepreneurs ( Ugwueze, Ike and Ugwu, 2022). This study's finding that students with higher alertness are better positioned to find and recognize opportunities and are more likely to start their businesses is a valuable insight that can inform efforts to promote entrepreneurship among young people. This highlights the importance of encouraging and developing entrepreneurial skills and mindsets in students and suggests that initiatives to promote entrepreneurship should focus not only on technical skills but also on fostering an entrepreneurial mindset.

Perceived behavioral control (PBC) (β = 0.208) was found to be the third most crucial factor affecting engineering students' EI, consistent with prior research studies ( Alnemer, 2021; Ambad, 2022; Lopez *et al*., 2021; Marmat, 2021; Naktiyok, Nur Karabey and CaglarGulluce, 2009). This suggests that engineering students with a higher level of PBC may have a greater intention to pursue entrepreneurship because they believe that they have the skills, knowledge, and resources necessary to start and run a successful venture. There could be several reasons why PBC was a significant predictor of EI among engineering students. One potential explanation for this relationship is that engineering students may have a strong sense of self-efficacy, which is a fundamental component of PBC ( Nguyen, Nguyen and Ba Le, 2022). Self-efficacy refers to an individual's belief in his or her ability to successfully perform a specific behavior. As engineering students gain technical and problem-solving skills during their studies, they may develop higher levels of self-efficacy than students from other fields ( Nguyen, Nguyen and Ba Le, 2022). The relationship between these constructs and entrepreneurial intention is supported by a growing body of empirical evidence. In a recent meta-analysis by Schlaegel and Koenig (2014), the authors analyzed 98 independent samples from 73 studies examining the antecedents of entrepreneurial intention. Their findings provide strong support for the positive relationship between entrepreneurial self-efficacy, perceived behavioral control, and entrepreneurial intention. Therefore, the finding that PBC is a crucial factor affecting engineering students' EI has significant implications for policymakers and educators interested in promoting entrepreneurship. This highlights the importance of creating an environment that supports self-efficacy and encourages the acquisition of the skills, knowledge, and resources necessary to pursue entrepreneurial activities.

The study's results revealed that attitude towards entrepreneurship (ATE) (β = 0.198) was the fourth most significant factor affecting students' EI, indicating a favorable ATE among students. This finding is consistent with previous studies conducted in diverse cultural and contextual settings, suggesting that the relationship between ATE and EI is likely to hold across various populations ( Al-Mamary *et al*., 2020; Aloulou, 2016; Maheshwari, 2021). In a cross-cultural context, Iakovleva *et al.* (2011) compared the determinants of entrepreneurial intention between students in Norway and Russia. The findings showed that, while the overall level of entrepreneurial intention differed between the two countries, the relationships between constructs such as perceived behavioral control, subjective norms, and entrepreneurial intention were consistent across the samples. This finding suggests that these relationships are robust in different cultural settings. The results suggest that engineering students desire to become their own bosses in the future and are more self-reliant. Promoting a positive attitude towards entrepreneurship may be an effective strategy for encouraging more students to consider entrepreneurship, which could have significant economic and social implications in the long run.

The need for achievement (NACH) (β = 0.127) is the fifth most important factor impacting engineering students' EI, suggesting that individuals with a high NACH are more likely to pursue entrepreneurial activities. This could be because individuals with a high NACH desire to set and achieve challenging goals, take calculated risks, and strive for independence and recognition, which are all critical components of entrepreneurial behavior. Moreover, previous research has consistently shown that NACH significantly predicts EI and behavior across various contexts and populations ( Bağış *et al*., 2022; Gürol and Atsan, 2006; Hansemark, 2003; Turker and SonmezSelcuk, 2009). This further supports the idea that individuals with high NACH are more likely to engage in entrepreneurial activities. McClelland (1961) identified the NACH as a critical factor in entrepreneurial success. This suggests that students with a higher NACH may be better equipped to handle the challenges and uncertainties of starting a new business, and are more likely to succeed. On the one hand, several studies have provided evidence supporting the positive influence of NACH on EI. Mukesh *et al.* (2019) found a significant predictive relationship between the NACH and EI among engineering students in India. Similarly, Chaudhary (2017) and Littunen (2000) reported a robust association between NACH and EI, suggesting that individuals with a high need for achievement are more inclined towards entrepreneurial activities because of their desire for accomplishment and autonomy.

However, some researchers have questioned the strength of the NACH-EI relationship. Davidsson and Wiklund (1999) argued that the role of NACH in predicting entrepreneurial behavior might be overstated, and its significance could be contingent on other factors, such as cultural context and industry type. This perspective is supported by Shapero and Sokol (1982), who highlight the critical role of situational and environmental factors in shaping entrepreneurial intentions, suggesting that the influence of NACH may vary significantly across different contexts.

This study found that risk tolerance (RT) (β = 0.054) was the least significant factor affecting engineering students' EI. This finding suggests that RT may not be a critical predictor of students' interest in pursuing entrepreneurial activities. This result is consistent with prior research by Ibidunni, Mozie and Ayeni (2020), Ilevbare *et al*. (2022) and Moraes, Iizuka and Pedro (2018). Lumpkin and Dess (1996) emphasized the importance of risk-taking propensity as a fundamental entrepreneurial trait across all levels. They suggest that individuals who are more willing to take calculated risks are more likely to become successful entrepreneurs. This view is consistent with the principles of Ajzen's theory of planned behaviour. This study's outcome may be attributed to various factors, such as cultural values and norms in India, which tend to prioritize stability and security over risk-taking behavior ( Chaudhary, 2017). Another possibility is that Indian engineering students view entrepreneurship as a low-risk pursuit due to established business networks, government support, and other factors ( Dubey, 2022). Nevertheless, it is essential to note that this finding does not imply that RT is not a crucial component of entrepreneurial behavior. Furthermore, a longitudinal study by Gielnik *et al.* (2017) followed a sample of university students in Germany over a three-year period. They found that entrepreneurial self-efficacy and perceptions of entrepreneurial opportunities were significant predictors of entrepreneurial intention, even after controlling for other factors, such as personality traits and demographic characteristics. This study demonstrated the robustness of these relationships. In contrast, taking calculated risks is often vital for entrepreneurial success. Educators and policymakers can encourage students to pursue entrepreneurial opportunities and develop successful businesses by promoting a culture of entrepreneurship, which highlights the importance of RT in a responsible and strategic manner.

The study found that the antecedents hypothesized regarding TPB accounted for 81% of the variance in students' EI. This percentage of variance is higher than the average explained variance reported in previous meta-analyses of TPB studies, indicating that the factors included in the study are highly relevant for predicting students' EI ( Mukesh *et al*., 2020). This finding suggests that the TPB model is a valuable framework for understanding and predicting students' EI.

Finally, the study found that the relationship between entrepreneurial intention (EI) and entrepreneurial behaviour (EB) is influenced by entrepreneurial motivation (EM). This research suggests that highly motivated students are more likely to engage in EB when they have a strong intention to become entrepreneurs. However, for students with low or medium EM, an increase in EI is likely to result in a corresponding EB increase. These results are consistent with the recommendations of Carsrud and Brännback (2010) and Lechuga Sancho, Ramos-Rodríguez and Frende Vega (2022), who highlight the importance of investigating the intention-behavior gap in entrepreneurship, an area that has received limited research attention.

### Practical implications

Unemployment is a significant problem in many developing nations, particularly India, where many educated individuals graduate from various academic institutions each year, but employment opportunities are scarce. To tackle this issue effectively, policymakers and educators must put more effort into recognizing and cultivating potential entrepreneurs. The outcomes of this study have essential practical applications for policymakers and educators in emerging economies.

The study found that ATE significantly influenced students' EI, which supports Hypothesis 1. Policymakers can use this information to develop policies and programs that encourage a positive ATE among engineering students, such as creating an entrepreneurial ecosystem on campuses that fosters innovation and creativity ( Dubey, 2022). This can include mentorship and networking programs, competition, and other experiential learning opportunities that expose students to entrepreneurship. Academicians can incorporate entrepreneurship education into engineering curricula to foster positive ATE. This can include courses covering topics such as opportunity recognition, business planning, marketing, and financial management as well as experiential learning such as internships, competitions, and incubation programs ( Mukesh *et al*., 2020).

SN is the most significant determinant of students' EI, supporting Hypothesis 2. This finding has practical implications for both policymakers and academics. For policymakers, this result suggests that efforts to promote entrepreneurship among engineering students should address the social and cultural norms surrounding entrepreneurship ( Boutaky and Sahib Eddine, 2022). This could involve creating awareness campaigns highlighting the impact of successful entrepreneurs on society and the benefits of entrepreneurship for individuals and communities. Additionally, policymakers could work towards establishing a supportive environment for entrepreneurs by providing access to financing, infrastructure, and other necessary resources for successful startups. For academics, this finding emphasizes the importance of developing programs and curricula that address the social and cultural norms related to entrepreneurship. Universities and engineering institutes can incorporate case studies and guest lecturers that showcase successful entrepreneurs and their contributions to society ( Bazkiaei *et al*., 2021). In addition, establishing mentorship programs that connect students with successful entrepreneurs in their field could be useful. Academics could also work towards identifying and overcoming cultural or social barriers to entrepreneurship among engineering students by providing resources and support to tackle these challenges.

This study supports hypothesis 3, indicating that PBC significantly influences students' EI. Therefore, policymakers and academicians can develop and promote programs that enhance engineering students' PBC towards entrepreneurship. This can include opportunities for students to participate in entrepreneurship training and education programs that focus on building their knowledge, skills, and self-efficacy regarding starting and managing a business ( Ambad, 2022; Lopez *et al*., 2021). To increase students' confidence in their ability to start and run a business, policymakers and academics can collaborate with industry partners to offer hands-on experiences and exposure to real-world entrepreneurial settings ( Marmat, 2021).

This study supports Hypothesis 4, indicating that engineering students' EA significantly and positively influences their EI. Policymakers and academicians can take practical steps to develop and enhance student EA by providing training programs and workshops that expose them to industry experts and successful entrepreneurs. These programs may include case studies and exercises designed to improve students' ability to identify entrepreneurial opportunities and take calculated risks. Additionally, academic institutions can encourage collaboration among students, faculty, and industry to foster an entrepreneurial culture on campus and provide students with more opportunities to hone their entrepreneurial skills ( Lim, Lee and Mamun, 2021). Policymakers can also promote innovation and entrepreneurship in the engineering sector by offering funding opportunities for startups and incentivizing businesses to invest in R&D ( Neneh, 2019). Policymakers and academics can contribute to developing the next generation of successful entrepreneurs in the engineering field by creating an environment that values entrepreneurship and innovation.

The NACH significantly influenced students' EI. This result supports hypothesis 5. To encourage more students to pursue entrepreneurship, policymakers should consider creating an environment that fosters achievement and success. This can be achieved by promoting and supporting entrepreneurial events and competitions, offering financial incentives and support to young entrepreneurs, and developing policies encouraging entrepreneurship ( Biswas and Verma, 2021). By creating an environment that rewards achievement, policymakers can help increase the number of engineering students who desire to start their own business. Academicians should also take note of this finding and use it to design and implement programs that foster a sense of achievement among engineering students. For example, entrepreneurship courses and programs can be developed that emphasize the importance of goal-setting, hard work, and perseverance in achieving success. These programs also offer opportunities for students to work on real-world entrepreneurial projects, which can help them build confidence and a sense of achievement. By providing engineering students with the tools and resources they need to succeed as entrepreneurs, academics can help increase the number of students pursuing their entrepreneurial careers.

RT significantly influenced students' EI, which supports Hypothesis 6. Policymakers can use this information to design policies and programs that encourage and support risk-taking behaviors among engineering students, such as providing access to funding, mentoring, and incubation programs that can help students mitigate and manage the risks associated with entrepreneurship ( Dubey, 2022). Additionally, policymakers can explore ways to incentivize industry collaborations and startup partnerships that expose engineering students to real-world experiences, which can increase their risk tolerance. Academics can use these findings to design and implement educational and training programs that help students develop and improve their risk-taking abilities ( Ilevbare *et al*., 2022). This can include integrating experiential learning opportunities into the engineering curriculum, such as business plan competitions and hackathons, where students can practice identifying and mitigating risks in a low-stake environment ( Ibidunni, Mozie and Ayeni, 2020). It can also offer courses and workshops on risk management and decision making to help students develop the skills and knowledge necessary to evaluate and manage the risks associated with entrepreneurship.

### Limitations and scope for future research

The limitations of this study suggest several avenues for future research. Researchers could expand the theoretical model used in this study to include environmental and external factors that may influence entrepreneurial intention and behavior. Furthermore, researchers could use qualitative or mixed-method approaches to gain a more in-depth understanding of the factors that contribute to entrepreneurial intention and behavior. Future studies could also include samples from other countries, such as South Asia, to provide cross-country data on the effectiveness of entrepreneurship education. Additionally, future research could explore the generalizability of the findings beyond India by including students of other nationalities. Longitudinal studies should be conducted to better understand the entrepreneurial journey of higher education students as they transition to becoming entrepreneurs. These studies could provide insights into the factors that influence the development and success of entrepreneurs as well as the challenges they face along the way.

## Ethical approval

Ethical approval was obtained on 04 June 2021 from the Kasturba Medical College and Kasturba Hospital Institutional Ethical Committee (registration number, IEC 235/2021). Completion of the questionnaire was also taken as the consent of the students to participate in the study.

## Data Availability

Figshare: Data set. https://doi.org/10.6084/m9.figshare.24203463 ( Nayak, 2023a). Figshare: Questionnaire. https://doi.org/10.6084/m9.figshare.24217848 ( Nayak, 2023b). The data are available under the terms of the Creative Commons Attribution 4.0 International license (CC-BY 4.0).

## References

[ref1] Ajzen I : The Theory of Planned Behavior. *Organ. Behav. Hum. Decis. Process.* 1991;50(2):179–211. 10.1016/0749-5978(91)90020-t

[ref5] Ajzen I Driver BL : Application of the Theory of Planned Behavior to Leisure Choice. *J. Leis. Res.* 1992;24(3):207–224. 10.1080/00222216.1992.11969889

[ref2] Ajzen I : Nature and Operation of Attitudes. *Annu. Rev. Psychol.* 2001;52(1):27–58. 10.1146/annurev.psych.52.1.27 11148298

[ref3] Ajzen I : Perceived behavioral control, self-efficacy, locus of control, and the theory of planned behavior. *J. Appl. Soc. Psychol.* 2002;32(4):665–683. 10.1111/j.1559-1816.2002.tb00236.x

[ref4] Ajzen I : The theory of planned behavior: Frequently asked questions. *Human Behavior and Emerging Technologies.* 2020;2(4):314–324. 10.1002/hbe2.195

[ref6] Al-Mamary YHS Abdulrab M Alwaheeb MA : Factors impacting entrepreneurial intentions among university students in Saudi Arabia: testing an integrated model of TPB and EO. *Educ. Train.* 2020;62(7/8):779–803. 10.1108/et-04-2020-0096

[ref7] Alnemer HA : Determinants of entrepreneurial intention among students of management stream in the Kingdom of Saudi Arabia. *Entrepreneurship Education.* 2021;4:425–445. 10.1007/s41959-021-00058-4

[ref8] Aloulou WJ : Predicting entrepreneurial intentions of final year Saudi university business students by applying the theory of planned behavior. *J. Small Bus. Enterp. Dev.* 2016;23(4):1142–1164. 10.1108/jsbed-02-2016-0028

[ref9] Ambad SNA : A Systematic Literature Review on Social Entrepreneurial Intention: Citation, Thematic Analyses and Future Research Directions. *Developments in Corporate Governance and Responsibility.* 2022;93–124. 10.1108/s2043-052320220000018006

[ref10] Ardichvili A Cardozo RN : A Model of The Entrepreneurial Opportunity Recognition Process. *Journal of Enterprising Culture.* 2000;08(02):103–119. 10.1142/s0218495800000073

[ref11] Armitage CJ Conner M : Efficacy of the Theory of Planned Behaviour: A meta-analytic review. *Br. J. Soc. Psychol.* 2001;40(4):471–499. 10.1348/014466601164939 11795063

[ref12] Autio E Keeley R Klofsten M : Entrepreneurial Intent among Students in Scandinavia and in the USA. *Enterp. Innov. Manag. Stud.* 2001;2(2):145–160. 10.1080/14632440110094632

[ref13] Bağış M Kryeziu L Kurutkan MN : Youth entrepreneurial intentions: a cross-cultural comparison. *Journal of Enterprising Communities: People and Places in the Global Economy.* 2022;17:769–792. 10.1108/jec-01-2022-0005

[ref133] Bandura A : Self-efficacy: toward a Unifying Theory of Behavioral Change. *Psychol. Rev.* 1977;84(2):191–215. 10.1037/0033-295X.84.2.191 847061

[ref15] Barba-Sánchez V Atienza-Sahuquillo C : Entrepreneurial behavior: Impact of motivation factors on decision to create a new venture. *InvestigacionesEuropeas de Dirección y Economía de la Empresa.* 2012;18(2):132–138. 10.1016/s1135-2523(12)70003-5

[ref14] Baum JR Locke EA : The relationship of entrepreneurial traits, skill, and motivation to subsequent venture growth. *J. Appl. Psychol.* 2004;89(4):587–598. 10.1037/0021-9010.89.4.587 15327346

[ref16] Bazkiaei HA Khan NU Irshad A-R : Pathways toward entrepreneurial intention among Malaysian universities’ students. *Bus. Process. Manag. J.* 2021;27:1009–1032. ahead-of-print (ahead-of-print). 10.1108/bpmj-01-2021-0021

[ref17] Bell R Cui J : Addressing progressive educational reforms: Fusing acquisition approaches and participation in Chinese entrepreneurship education. *The International Journal of Management Education.* 2023;21(1):100748. 10.1016/j.ijme.2022.100748

[ref18] Bird BJ : *Entrepreneur behavior.* Glenview, IL: Scott, Foresman and Company;1989.

[ref19] Biswas A Verma RK : Engine of entrepreneurial intentions: revisiting personality traits with entrepreneurial education. *BIJ.* 2021;29:2019–2044. ahead-of-print (ahead-of-print). 10.1108/bij-11-2020-0607

[ref20] Boutaky S Sahib Eddine A : Determinants of entrepreneurial intention among scientific students: A social cognitive theory perspective. *Ind. High. Educ.* 2022;37:279–293. 10.1177/09504222221120750

[ref21] Boyd NG Vozikis GS : The Influence of Self-Efficacy on the Development of Entrepreneurial Intentions and Actions. *Entrep. Theory Pract.* 1994;18(4):63–77. 10.1177/104225879401800404

[ref42] Brás GR Daniel A Fernandes CI : The effect of proximal personality traits on entrepreneurial intention among higher education students. 2023. 10.1108/ijis-10-2022-0198

[ref22] Brockhaus RH : Risk Taking Propensity of Entrepreneurs. *Acad. Manag. J.* 1980;23(3):509–520. 10.5465/255515

[ref991] Busenitz LW Lau C-M : A cross-cultural cognitive model of new venture creation. *Entrepreneurship Theory and Practice* .1996;20(4):25탖40. 10.1177/104225879602000403

[ref23] CantúCavada M Bobek V Maček A : Motivation Factors for Female Entrepreneurship in Mexico. *Entrepreneurial Business and Economics Review.* 2017;5(3):133–148. 10.15678/eber.2017.050307

[ref24] Carsrud A Brännback M : Entrepreneurial Motivations: What Do We Still Need to Know? *J. Small Bus. Manag.* 2010;49(1):9–26. 10.1111/j.1540-627x.2010.00312.x

[ref25] Carsrud AL Olm KW Thomas JB : Predicting entrepreneurial success: effects of multi-dimensional achievement motivation, levels of ownership, and cooperative relationships. *Entrep. Reg. Dev.* 1989;1(3):237–244. 10.1080/08985628900000020

[ref26] Chatterjee N Das N Srivastava NK : A structural model assessing key factors affecting women’s entrepreneurial success. *Journal of Entrepreneurship in Emerging Economies.* 2019;11(1):122–151. 10.1108/jeee-08-2016-0030

[ref27] Chaudhary R : Demographic factors, personality and entrepreneurial inclination. *Educ. Train.* 2017;59(2):171–187. 10.1108/et-02-2016-0024

[ref28] Chhabra S Raghunathan R Rao NVM : The antecedents of entrepreneurial intention among women entrepreneurs in India. *Asia Pacific Journal of Innovation and Entrepreneurship.* 2020;14(1):76–92. 10.1108/apjie-06-2019-0034

[ref29] Conner M Armitage CJ : Extending the theory of planned behavior: A review and avenues for further research. *Journal of Applied Social Psychology* .1998;28(15):1429탖1464. 10.1111/j.1559-1816.1998.tb01685.x

[ref30] Davidsson P Wiklund J : Suitable approaches for studying small firm growth: the role of entrepreneurship and small and medium enterprises. *Proceedings of the 44th ICSB World Conference, Naples.* 1999 June;20–23

[ref31] Dawson C Henley A : ‘Push’ versus ‘pull’ entrepreneurship: an ambiguous distinction? *Int. J. Entrep. Behav. Res.* 2012;18(6):697–719. 10.1108/13552551211268139

[ref41] Di Stefano G Ruggieri S Bonfanti RC : Entrepreneurship on Social Networking Sites: The Roles of Attitude and Perceived Usefulness. 2023;13(4):323–323. 10.3390/bs13040323 37102838 PMC10136023

[ref32] Dinis A Paço A do Ferreira J : Psychological characteristics and entrepreneurial intentions among secondary students. *Educ. Train.* 2013;55(8/9):763–780. 10.1108/et-06-2013-0085

[ref33] Douglas EJ Shepherd DA : Self-Employment as a Career Choice: Attitudes, Entrepreneurial Intentions, and Utility Maximization. *Entrep. Theory Pract.* 2002;26(3):81–90. 10.1177/104225870202600305

[ref34] Dubey P : The effect of entrepreneurial characteristics on attitude and intention: an empirical study among technical undergraduates. *Journal of Business and Socio-economic Development.* 2022. 10.1108/jbsed-09-2021-0117

[ref135] Dubey P Sahu KK : Examining the effects of demographic, social and environmental factors on entrepreneurial intention. *Manag. Matters.* 2022;19(1):91–108. 10.1108/manm-12-2021-0006

[ref35] Echchabi A Ayedh AM Omar MMS : Entrepreneurial intention among female university students in Oman. *J. Int. Bus. Entrep. Dev.* 2020;12(4):280. 10.1504/jibed.2020.10032496

[ref36] Esfandiar K Sharifi-Tehrani M Pratt S : Understanding entrepreneurial intentions: A developed integrated structural model approach. *J. Bus. Res.* 2019;94:172–182. 10.1016/j.jbusres.2017.10.045

[ref37] Farooq MS Salam M ur Rehman S : Impact of support from social network on entrepreneurial intention of fresh business graduates. *Educ. Train.* 2018;60(4):335–353. 10.1108/et-06-2017-0092

[ref38] Fornell C Larcker DF : Evaluating Structural Equation Models with Unobservable Variables and Measurement Error. *J. Mark. Res.* 1981;18(1):39–50. 10.1177/002224378101800104

[ref39] Gibson S Harris M Ghouse SM : Role of gender and exposure on entrepreneurial attitudes of Omani university students. *J. Int. Bus. Entrep. Dev.* 2021;1(1):1. 10.1504/jibed.2021.10031623

[ref140] Gielnik MM Zacher H Frese M : Focus on opportunities as a mediator of the relationship between business owners’ age and venture growth. *J. Bus. Ventur.* 2012;27(1),127–142. 10.1016/j.jbusvent.2010.05.002

[ref40] Gieure C Benavides-Espinosa M d M Roig-Dobón S : The entrepreneurial process: The link between intentions and behavior. *J. Bus. Res.* 2020;112:541–548. 10.1016/j.jbusres.2019.11.088

[ref43] Gull N Asghar M Aleem Ahmed Q : Entrepreneurial orientation and international performance of born global firms: the mediating role of entrepreneurial competencies. *Vilakshan - XIMB Journal of Management.* 2021;18(2):122–137. 10.1108/xjm-06-2020-0009

[ref45] Gürol Y Atsan N : Entrepreneurial characteristics amongst university students. *Educ. Train.* 2006;48(1):25–38. 10.1108/00400910610645716

[ref44] Gultom S Dalle J Restu : THE INFLUENCE OF ATTITUDE AND SUBJECTIVE NORM ON CITIZEN’S INTENTION TO USE E-GOVERNMENT SERVICES. *Journal of Security and Sustainability Issues.* 2020;9(M). 10.9770/jssi.2020.9.m(14)

[ref136] Gupta VK Bhawe NM : The Influence of Proactive Personality and Stereotype Threat on Women’s Entrepreneurial Intentions. *J. Leadersh. Organ. Stud.* 2007;13(4):73–85. 10.1177/10717919070130040901

[ref46] Hair JF Black WC Babin BJ : *Multivariate data analysis.* Englewood Cliffs, NJ: Prentice Hall;2010.

[ref47] Hansemark OC : Need for achievement, locus of control and the prediction of business start-ups: A longitudinal study. *J. Econ. Psychol.* 2003;24(3):301–319. 10.1016/s0167-4870(02)00188-5

[ref48] Hassan A Anwar I Saleem A : Nexus between entrepreneurship education, motivations, and intention among Indian university students: The role of psychological and contextual factors. *Ind. High. Educ.* 2021;36:539–555. 10.1177/09504222211053262

[ref49] Helmreich RL Sawin LL Carsrud AL : The honeymoon effect in job performance: Temporal increases in the predictive power of achievement motivation. *J. Appl. Psychol.* 1986;71(2):185–188. 10.1037/0021-9010.71.2.185 11538825

[ref50] Hoang G Le TTT Tran AKT : Entrepreneurship education and entrepreneurial intentions of university students in Vietnam: the mediating roles of self-efficacy and learning orientation. *Educ. Train.* 2020;63(1):115–133. 10.1108/et-05-2020-0142

[ref51] Hofstede G : Dimensionalizing Cultures: The Hofstede Model in Context. *Online Readings in Psychology and Culture.* 2011;2(1):1–26. 10.9707/2307-0919.1014

[ref52] Hosen M Ogbeibu S Lim WM : Knowledge sharing behavior among academics: Insights from theory of planned behavior, perceived trust and organizational climate. *J. Knowl. Manag.* 2022;27:1740–1764. 10.1108/jkm-02-2022-0140

[ref137] Hsu DK Wiklund J Anderson SE : Entrepreneurial exit intentions and the business-family interface. *J. Bus. Ventur.* 2016;31(6):613–627. 10.1016/j.jbusvent.2016.08.001

[ref53] Hu L Bentler PM : Cutoff criteria for fit indexes in covariance structure analysis: Conventional criteria versus new alternatives. *Struct. Equ. Model. Multidiscip. J.* 1999;6(1):1–55. 10.1080/10705519909540118

[ref54] Hu R Wang L Zhang W : Creativity, Proactive Personality, and Entrepreneurial Intention: The Role of Entrepreneurial Alertness. *Front. Psychol.* 2018;9. 10.3389/fpsyg.2018.00951 29962985 PMC6011088

[ref139] Iakovleva T Kolvereid L Stephan U : Entrepreneurial intentions in developing and developed countries. *Education + Training.* 2011;53(5):353–370. 10.1108/00400911111147686

[ref55] Ibidunni AS Mozie D Ayeni AWAA : Entrepreneurial characteristics amongst university students: insights for understanding entrepreneurial intentions amongst youths in a developing economy. *Educ. Train.* 2020;63:71–84. ahead-of-print (ahead-of-print). 10.1108/et-09-2019-0204

[ref56] Ilevbare FM Ilevbare OE Adelowo CM : Social support and risk-taking propensity as predictors of entrepreneurial intention among undergraduates in Nigeria. *Asia Pacific Journal of Innovation and Entrepreneurship.* 2022;16:90–107. 10.1108/apjie-02-2022-0010

[ref57] Iqbal N Khan A Gill AS : Nexus between sustainable entrepreneurship and environmental pollution: evidence from developing economy. *Environ. Sci. Pollut. Res.* 2020;27:36242–36253. 10.1007/s11356-020-09642-y 32556976

[ref58] Khan R Anwar I Thoudam P : Entrepreneurial intention among female university students: examining the moderating role of entrepreneurial education. *J. Int. Bus. Entrep. Dev.* 2020;12(4):217. 10.1504/jibed.2020.10032497

[ref60] Kolvereid L : Prediction of Employment Status Choice Intentions. *Entrep. Theory Pract.* 1996;21(1):47–58. 10.1177/104225879602100104

[ref61] Krueger NF Carsrud AL : Entrepreneurial intentions: Applying the theory of planned behaviour. *Entrep. Reg. Dev.* 1993;5(4):315–330. 10.1080/08985629300000020

[ref62] Krueger NF Reilly MD Carsrud AL : Competing models of entrepreneurial intentions. *J. Bus. Ventur.* 2000;15(5-6):411–432. 10.1016/s0883-9026(98)00033-0

[ref63] Kumar R Shukla S : A theory-based approach to model entrepreneurial intentions: exploring the role of creativity, proactive personality and passion. *Higher Education, Skills and Work-Based Learning.* 2023;13:355–370. 10.1108/heswbl-02-2022-0036

[ref64] Kuratko DF Fisher G Bloodgood JM : The paradox of new venture legitimation within an entrepreneurial ecosystem. *Small Bus. Econ.* 2017;49(1):119–140. 10.1007/s11187-017-9870-x

[ref65] Lechuga Sancho MP Ramos-Rodríguez AR Frende Vega M d l Á : The influence of university entrepreneurship-oriented training in the transformation of intentions into new businesses. *The International Journal of Management Education.* 2022;20(2):100631. 10.1016/j.ijme.2022.100631

[ref66] Lee V-H Ooi K-B Chong AY-L : A structural analysis of greening the supplier, environmental performance and competitive advantage. *Prod. Plan. Control.* 2013;26(2):116–130. 10.1080/09537287.2013.859324

[ref67] Lee V-H Ooi K-B Chong AY-L : Creating technological innovation via green supply chain management: An empirical analysis. *Expert Syst. Appl.* 2014;41(16):6983–6994. 10.1016/j.eswa.2014.05.022

[ref68] Li C Murad M Shahzad F : Entrepreneurial Passion to Entrepreneurial Behavior: Role of Entrepreneurial Alertness, Entrepreneurial Self-Efficacy and Proactive Personality. *Front. Psychol.* 2020;11. 10.3389/fpsyg.2020.01611 32973593 PMC7468520

[ref69] Lim W Lee Y Mamun AA : Delineating competency and opportunity recognition in the entrepreneurial intention analysis framework. *Journal of Entrepreneurship in Emerging Economies.* 2021;15:212–232. ahead-of-print (ahead-of-print). 10.1108/jeee-02-2021-0080

[ref70] Liñán F Chen Y-W : Development and Cross-Cultural Application of a Specific Instrument to Measure Entrepreneurial Intentions. *Entrep. Theory Pract.* 2009;33(3):593–617. 10.1111/j.1540-6520.2009.00318.x

[ref71] Liñán F Rodríguez-Cohard JC Rueda-Cantuche JM : Factors affecting entrepreneurial intention levels: a role for education. *Int. Entrep. Manag. J.* 2010;7(2):195–218. 10.1007/s11365-010-0154-z

[ref72] Littunen H : Entrepreneurship and the characteristics of the entrepreneurial personality. *Int. J. Entrep. Behav. Res.* 2000;6(6):295–310. 10.1108/13552550010362741

[ref73] Lockyer J George S : What women want: barriers to female entrepreneurship in the West Midlands. *Int. J. Gend. Entrep.* 2012;4(2):179–195. 10.1108/17566261211234661

[ref74] Lopez T Alvarez C Martins I : Students’ perception of learning from entrepreneurship education programs and entrepreneurial intention in Latin America. *Academia RevistaLatinoamericana de Administración.* 2021;34(3):419–444. 10.1108/arla-07-2020-0169

[ref75] Lu H Wang J : Entrepreneurial Intention of Two Patterns of Planned Behaviour and Alertness: Empirical Evidence in China. *The Journal of Asian Finance, Economics and Business.* 2018;5(2):63–72. 10.13106/jafeb.2018.vol5.no2.63

[ref76] Lumpkin GT Dess GG : Clarifying the Entrepreneurial Orientation Construct and Linking It To Performance. *Acad. Manag. Rev.* 1996;21(1):135–172. 10.2307/258632

[ref77] Maheshwari G : Factors influencing entrepreneurial intentions the most for university students in Vietnam: educational support, personality traits or TPB components? *Educ. Train.* 2021;63:1138–1153. 10.1108/et-02-2021-0074

[ref78] Mahmoodi G RasoolyKalamaki F Yazdani-Charati J : A Model for Organizational Entrepreneurship with Entrepreneurial Personality Traits Approach of District 1 Iran Teaching Hospitals. *Jundishapur J. Health Sci.* 2023;15(1). 10.5812/jjhs-134220

[ref80] Mannino G Schiera M : La famigliaomogenitorialeoggi: pregiudizio per lo sviluppo del minore? Un’analisidellaletteratura dal 2000 al 2015. *MALTRATTAMENTO E ABUSO ALL’INFANZIA.* 2017;19(3):87–109. 10.3280/mal2017-003006

[ref81] Marmat G : Predicting intention of business students to behave ethically in the Indian context: from the perspective of Theory of Planned Behaviour. *Higher Education, Skills and Work-Based Learning.* 2021;12:437–458. 10.1108/heswbl-05-2021-0090

[ref82] Marques AC Fuinhas JA : Is renewable energy effective in promoting growth? *Energy Policy.* 2012;46:434–442. 10.1016/j.enpol.2012.04.006

[ref83] McClelland DC : Entrepreneurial behavior. *The achieving society.* 1961;205–258. 10.1037/14359-006

[ref84] McClelland DC : N achievement and entrepreneurship: A longitudinal study. *J. Pers. Soc. Psychol.* 1965;1(4):389–392. 10.1037/h0021956 14328753

[ref85] McMullen JS Shepherd DA : Entrepreneurial Action And The Role Of Uncertainty In The Theory Of The Entrepreneur. *Acad. Manag. Rev.* 2006;31(1):132–152. 10.5465/amr.2006.19379628

[ref86] Minola T Criaco G Obschonka M : Age, culture, and self-employment motivation. *Small Bus. Econ.* 2015;46(2):187–213. 10.1007/s11187-015-9685-6

[ref87] Mitchell RK Busenitz L Lant T : Toward a Theory of Entrepreneurial Cognition: Rethinking the People Side of Entrepreneurship Research. *Entrep. Theory Pract.* 2002;27(2):93–104. 10.1111/1540-8520.00001

[ref88] Montiel Campos H : Impact of entrepreneurial passion on entrepreneurial orientation with the mediating role of entrepreneurial alertness for technology-based firms in Mexico. *J. Small Bus. Enterp. Dev.* 2017;24(2):353–374. 10.1108/jsbed-10-2016-0166

[ref89] Moraes GHSM de Iizuka ES Pedro M : Effects of Entrepreneurial Characteristics and University Environment on Entrepreneurial Intention. *Revista de AdministraçãoContemporânea.* 2018;22(2):226–248. 10.1590/1982-7849rac2018170133

[ref90] Mukesh HV Pillai KR Mamman J : Action-embedded pedagogy in entrepreneurship education: an experimental enquiry. *Stud. High. Educ.* 2019;45:1679–1693. 10.1080/03075079.2019.1599848

[ref91] Mukesh HV Prabhu N Koodamara NK : Entrepreneurial potential of students of MBA and engineering schools in the Indian context: roles of leadership and achievement motivation. *Journal of Applied Research in Higher Education.* 2020;13(3):782–810. 10.1108/jarhe-01-2020-0025

[ref92] Mukesh HV Rao AS Rajasekharan Pillai K : Entrepreneurial Potential and Higher Education System in India. *J. Entrep.* 2018;27(2):258–276. 10.1177/0971355718781275

[ref93] Naktiyok A Nur Karabey C CaglarGulluce A : Entrepreneurial self-efficacy and entrepreneurial intention: the Turkish case. *Int. Entrep. Manag. J.* 2009;6(4):419–435. 10.1007/s11365-009-0123-6

[ref95] Nayak P : Questionnaire. figshare. *Figure.* 2023b. 10.6084/m9.figshare.24217848.v1

[ref94] Nayak P : Data set.[Dataset]. *figshare.* 2023a. 10.6084/m9.figshare.24203463.v1

[ref96] Neneh BN : From entrepreneurial intentions to behavior: The role of anticipated regret and proactive personality. *J. Vocat. Behav.* 2019;112:311–324. 10.1016/j.jvb.2019.04.005

[ref97] Nguyen CQ Nguyen AMT Ba Le L : Using partial least squares structural equation modeling (PLS-SEM) to assess the effects of entrepreneurial education on engineering students’s entrepreneurial intention. *Cogent Education.* 2022;9(1). 10.1080/2331186x.2022.2122330

[ref99] Ouni S Boujelbene Y : The mediating role of big five traits and self-efficacy on the relationship between entrepreneurship education and entrepreneurial behavior: Study of Tunisian university graduate employees. *Eval. Program Plann.* 2023;100:102325. 10.1016/j.evalprogplan.2023.102325 37290210

[ref100] Panda NM : What Brings Entrepreneurial Success in a Developing Region? *J. Entrep.* 2000;9(2):199–212. 10.1177/097135570000900204

[ref101] Pandit D Joshi MP Tiwari SR : Examining Entrepreneurial Intention in Higher Education: An Exploratory Study of College Students in India. *J. Entrep.* 2018;27(1):25–46. 10.1177/0971355717738595

[ref102] Passah KH Panda NM : A structural relationship model of gender-role orientation and entrepreneurial intention: examining the mediating effect of motivational antecedents in Northeast India. *Int. J. Gend. Entrep.* 2021;14:167–187. ahead-of-print (ahead-of-print). 10.1108/ijge-03-2021-0039

[ref103] Rauch A Frese M : Let’s put the person back into entrepreneurship research: A meta-analysis on the relationship between business owners’ personality traits, business creation, and success. *Eur. J. Work Organ. Psy.* 2007;16(4):353–385. 10.1080/13594320701595438

[ref104] Robert FC Frey LM Sisodia GS : Village development framework through self-help-group entrepreneurship, microcredit, and anchor customers in solar microgrids for cooperative sustainable rural societies. *J. Rural. Stud.* 2021;88(1):432–440. 10.1016/j.jrurstud.2021.07.013

[ref106] Ruiz-Dotras E Lladós-Masllorens J : Entrepreneurial Self-efficacy and Financial and Calculation Skills Can Shape Different Profiles of Venture Intentions. *J. Entrep.* 2022;31(1):153–183. 10.1177/09713557211069319

[ref107] Ruizalba Robledo JL VallespínArán M Martin Sanchez V : The moderating role of gender on entrepreneurial intentions: A TPB perspective. *Intangible Capital.* 2015;11(1). 10.3926/ic.557

[ref108] Ryan RM Deci EL : Self-Determination Theory and the Facilitation of Intrinsic Motivation, Social Development, and Well-Being. *Am. Psychol.* 2000;55(1):68–78. 10.1037/0003-066X.55.1.68 11392867

[ref138] Schlaegel C Koenig M : Determinants of Entrepreneurial Intent: A Meta-Analytic Test and Integration of Competing Models. *Entrep. Theory Pr.* 2014;38(2):291–332 10.1111/etap.12087

[ref110] Sexton DL Smilor RW : The Art and Science of Entrepreneurship, University of Illinois at Urbana-Champaign’s Academy for Entrepreneurial Leadership Historical Research Reference in Entrepreneurship. 1986. Reference Source

[ref111] Shamsudeen K Keat OY Hassan H : Entrepreneurial Success within the Process of Opportunity Recognition and Exploitation: An Expansion of Entrepreneurial Opportunity Recognition Model. *Int. Rev. Manag. Mark.* 2017;7(1):107–111.

[ref112] Shapero A Sokol L : *The Social Dimensions of Entrepreneurship.* Englewood Cliffs:1982. Reference Source

[ref113] Short JC Ketchen DJ Shook CL : The Concept of ‘Opportunity’ in Entrepreneurship Research: Past Accomplishments and Future Challenges. *J. Manag.* 2009;36(1):40–65. 10.1177/0149206309342746

[ref114] Shrivastava U Acharya SR : Entrepreneurship education intention and entrepreneurial intention amongst disadvantaged students: an empirical study. *Journal of Enterprising Communities: People and Places in the Global Economy.* 2020;15:313–333. ahead-of-print (ahead-of-print). 10.1108/jec-04-2020-0072

[ref115] Smilor RW Kuhn RL : *Managing take-off in fast growth companies: innovations in entrepreneurial firms.* Santa Barbara: Praeger publishers;1986.

[ref116] Sobaih AEE Elshaer IA : Risk-Taking, Financial Knowledge, and Risky Investment Intention: Expanding Theory of Planned Behavior Using a Moderating-Mediating Model. *Mathematics.* 2023;11(2):453. 10.3390/math11020453

[ref117] Tang J : Environmental munificence for entrepreneurs: entrepreneurial alertness and commitment. *Int. J. Entrep. Behav. Res.* 2008;14(3):128–151. 10.1108/13552550810874664

[ref118] Tang J Kacmar KM(M) Busenitz L : Entrepreneurial alertness in the pursuit of new opportunities. *J. Bus. Ventur.* 2012;27(1):77–94. 10.1016/j.jbusvent.2010.07.001

[ref119] Thomas O : Entrepreneurship education: Which educational elements influence entrepreneurial intention? *Ind. High. Educ.* 2022;37:328–344. 10.1177/09504222221121065

[ref120] Thoudam P Saleem I Anwar I : Traits and entrepreneurial intention: testing the mediating role of entrepreneurial attitude and self-efficacy. *J. Int. Bus. Entrep. Dev.* 2021;13(1):40. 10.1504/jibed.2021.10034433

[ref121] Turker D SonmezSelcuk S : Which factors affect entrepreneurial intention of university students? *J. Eur. Ind. Train.* 2009;33(2):142–159. 10.1108/03090590910939049

[ref134] Tversky A Kahneman D : Judgment under Uncertainty: Heuristics and Biases. *Science.* 1974;185(4157):1124–1131. 10.1126/science.185.4157.1124 17835457

[ref122] Ugwueze AU Ike OO Ugwu L : Responding to social change: innovativeness, entrepreneurial alertness, and entrepreneurial intention in Nigeria: the role of family support. *Entrepreneurship Education.* 2022;5:465–485. 10.1007/s41959-022-00082-y

[ref123] Veleva V : The role of entrepreneurs in advancing sustainable lifestyles: Challenges, impacts, and future opportunities. *J. Clean. Prod.* 2020;283(1):124658. 10.1016/j.jclepro.2020.124658 33078048 PMC7556791

[ref124] Voutsina K Papagiannakis G Lioukas S : Entrepreneurial intentions in times of economic recession: the dual role of environment. *J. Int. Bus. Entrep. Dev.* 2022;14(3):265. 10.1504/jibed.2022.126949

[ref125] Xin B Ma X : Gamifying Online Entrepreneurship Education and Digital Entrepreneurial Intentions: An Empirical Study. *Entertainment Computing.* 2023;46:100552. 10.1016/j.entcom.2023.100552

[ref126] Yap SF Othman MN Wee YG : The fallacy of one-dimensional theory of planned behaviour structure in predicting health behaviour. *International Journal of Behavioural and Healthcare Research.* 2013;4(1):26. 10.1504/ijbhr.2013.054516

[ref127] Yar Hamidi D Wennberg K Berglund H : Creativity in entrepreneurship education. *J. Small Bus. Enterp. Dev.* 2008;15(2):304–320. 10.1108/14626000810871691

[ref128] Yasir N Babar M Mehmood HS : The Environmental Values Play a Role in the Development of Green Entrepreneurship to Achieve Sustainable Entrepreneurial Intention. *Sustainability.* 2023;15(8):6451. 10.3390/su15086451

[ref129] Zahid H Haji Din B : Determinants of Intention to Adopt E-Government Services in Pakistan: An Imperative for Sustainable Development. *Resources.* 2019;8(3):128. 10.3390/resources8030128

[ref130] Zainuddin MN Mukhtar D : Postgraduate entrepreneurship education: can entrepreneurial passion be developed? *Journal of Entrepreneurship in Emerging Economies.* 2022. 10.1108/jeee-06-2021-0237

[ref131] Zellweger T Sieger P Halter F : Should I stay or should I go? Career choice intentions of students with family business background. *J. Bus. Ventur.* 2011;26(5):521–536. 10.1016/j.jbusvent.2010.04.001

[ref132] Zgheib P : Multi-level framework of push-pull entrepreneurship: comparing American and Lebanese women. *Int. J. Entrep. Behav. Res.* 2018;24(3):768–786. 10.1108/ijebr-12-2015-0314

