# Supplementary material for: The moderating effect of entrepreneurial motivation on the relationship between entrepreneurial intention and behaviour: An extension of the theory of planned behaviour on emerging economy
Source: F1000Res. 2024 Jul 1;12:1585. Originally published 2023 Dec 14. [Version 2] doi: 10.12688/f1000research.140675.2 (PMC11231634; doi:10.12688/f1000research.140675.2)
Supplement: Peer review history

# Reviewer report: Reviewer response for version 2

**DOI:** 10.5256/f1000research.165981.r297545

**Published:** 2024-07-11

**Authors:** Pradeep Kumar Mohanty

**Affiliations:** 1 School of Management, Bennett University, Greater Noida, Uttar pradesh, India

Dear Editor,

 The authors have done an excellent job with their revisions. I am happy to see the outcome of this research work. I congratulate the authors.

Is the work clearly and accurately presented and does it cite the current literature?

Yes

If applicable, is the statistical analysis and its interpretation appropriate?

Yes

Are all the source data underlying the results available to ensure full reproducibility?

Yes

Is the study design appropriate and is the work technically sound?

Yes

Are the conclusions drawn adequately supported by the results?

Yes

Are sufficient details of methods and analysis provided to allow replication by others?

Yes

Reviewer Expertise:

Customer Satisfaction & Loyalty, Purchase Intention, Behavior Buyer-Seller relationship, CSR, Consumer behaviour

I confirm that I have read this submission and believe that I have an appropriate level of expertise to confirm that it is of an acceptable scientific standard.

# Reviewer report: Reviewer response for version 2

**DOI:** 10.5256/f1000research.165981.r297546

**Published:** 2024-07-08

**Authors:** Wisuwat Wannamakok

**Affiliations:** 1 Chaing Mai University, Chiang Mai, Thailand

Thank you for the opportunity to review this work. Your effort in revising the paper has significantly improved and theoretical sounds. I only have one more suggestion. 

 Please separate the theoretical and practical implication to have a clearer picture of the contribution of this work.

 Good luck and keep on the good work.

Is the work clearly and accurately presented and does it cite the current literature?

Partly

If applicable, is the statistical analysis and its interpretation appropriate?

Yes

Are all the source data underlying the results available to ensure full reproducibility?

Yes

Is the study design appropriate and is the work technically sound?

Yes

Are the conclusions drawn adequately supported by the results?

Partly

Are sufficient details of methods and analysis provided to allow replication by others?

Yes

Reviewer Expertise:

Entrepreneurship

I confirm that I have read this submission and believe that I have an appropriate level of expertise to confirm that it is of an acceptable scientific standard.

# Reviewer report: Reviewer response for version 1

**DOI:** 10.5256/f1000research.154059.r255298

**Published:** 2024-03-27

**Authors:** Wisuwat Wannamakok

**Affiliations:** 1 Chaing Mai University, Chiang Mai, Thailand

Thank you for the opportunity to review this research paper.

 The introduction provided is well-defined and comprehensive. It starts by contextualizing entrepreneurship's importance in economic development, especially in emerging economies like India, and highlights the increasing interest in entrepreneurship among the Indian youth. 

 Your literature review provides a detailed exploration of the theoretical constructs related to entrepreneurial intention (EI), including attitude towards entrepreneurship (ATE), subjective norm (SN), perceived behavioral control (PBC), entrepreneurial alertness (EA), need for achievement (NACH), and risk tolerance (RT).  To further enrich your literature review, consider the following suggestions:

 Include Recent Studies: Look for studies published within the last five years to ensure your review incorporates the latest research findings.

 Integration of Theoretical Perspectives: While you've done a thorough job of summarizing the literature related to each construct, consider integrating different theoretical perspectives that provide a holistic understanding of entrepreneurial intention formation. For example, besides the Theory of Planned Behavior (TPB), you can explore other relevant theories such as Social Cognitive Theory, Theory of Entrepreneurial Event, or Dual Process Theory, which offer complementary insights into entrepreneurial decision-making.

 Discussion of Empirical Findings: While you've mentioned some empirical studies supporting the relationships between the constructs and entrepreneurial intention, consider providing a more in-depth discussion of the empirical evidence. Highlight the methodologies used, sample characteristics, and key findings of these studies to illustrate the robustness of the relationships in different contexts.

 Critique and Controversies: Discuss any controversies or debates in the literature regarding the relationships between the constructs and entrepreneurial intention. For instance, you briefly mentioned conflicting findings regarding the influence of need for achievement (NACH) on entrepreneurial intention. Expanding on these debates and discussing possible explanations for conflicting results can add depth to your literature review.

 Cultural Considerations: Given that your study focuses on engineering students in India, it's important to discuss how cultural factors may shape attitudes, norms, and behaviors related to entrepreneurship in the Indian context. Explore literature that examines cultural dimensions (e.g., collectivism, uncertainty avoidance) and their impact on entrepreneurial intention formation.

 Please add theoretical implications.

 Lastly, the content is grammatically correct overall. However, few minor issues that could be improved for clarity and coherence. Therefore, proofreading is suggested.

Is the work clearly and accurately presented and does it cite the current literature?

Partly

If applicable, is the statistical analysis and its interpretation appropriate?

Yes

Are all the source data underlying the results available to ensure full reproducibility?

Yes

Is the study design appropriate and is the work technically sound?

Yes

Are the conclusions drawn adequately supported by the results?

Partly

Are sufficient details of methods and analysis provided to allow replication by others?

Yes

Reviewer Expertise:

Entrepreneurship

I confirm that I have read this submission and believe that I have an appropriate level of expertise to confirm that it is of an acceptable scientific standard, however I have significant reservations, as outlined above.

# Response

**Published:** 2024-04-27

**Authors:** Madhukara Nayak

**Affiliations:** Mechanical, Shri Madhwa Vadiraja Institute of Technology & Management, Udupi, Karnataka, India

Dear Sir/Madam,

 I am writing to express my gratitude for the time and effort you have invested in reviewing our manuscript, entitled “ **The moderating effect of entrepreneurial motivation on the relationship between entrepreneurial intention and behaviour: An extension of the theory of planned behaviour on emerging economy**”. Your expertise and thoughtful feedback have been invaluable in helping us to improve the quality and clarity of our work.

 We appreciate your insightful comments and suggestions, which have helped us to address the issues raised and to strengthen our manuscript. Your efforts have greatly contributed to the further development and refinement of our research. Once again, thank you for your time and expertise in reviewing our manuscript. We are grateful for your input and look forward to the possibility of working with you again in the future. We have given the review response for each questions in Tabular form.

 Sincerely,

 Authors

**Comments & Authors Answers**

**Reviewer 2 Comments & Responses (Dr Wisuwat Wannamakok)**

**Comments: Include Recent Studies: Look for studies published within the last five years to ensure your review incorporates the latest research findings.**

**Responses:** *We have considered your valuable suggestions and incorporated the necessary changes into the in the revised manuscript. We have added latest relevant literature in the introduction and review of literature section and discussion section.*

**Comments: Integration of Theoretical Perspectives:** **While you've done a thorough job of summarizing the literature related to each construct, consider integrating different theoretical perspectives that provide a holistic understanding of entrepreneurial intention formation. For example, besides the Theory of Planned Behavior (TPB), you can explore other relevant theories such as Social Cognitive Theory, Theory of Entrepreneurial Event, or Dual Process Theory, which offer complementary insights into entrepreneurial decision-making.**

**Responses:** *We have considered your valuable suggestions and incorporated the necessary changes into the in the revised manuscript. We have incorporated these changes in the introduction section with the highlights. The current research article has been focused on theory of planned behavior and we have mentioned the usage these theories in other contexts. And also, we have highlighted usage of various other theories in entrepreneurial decision-making.*

**Comments:** **Discussion of Empirical Findings: While you've mentioned some empirical studies supporting the relationships between the constructs and entrepreneurial intention, consider providing a more in-depth discussion of the empirical evidence. Highlight the methodologies used, sample characteristics, and key findings of these studies to illustrate the robustness of the relationships in different contexts.**

**Responses:** *We have considered your valuable suggestions and incorporated the necessary changes into the in the revised manuscript. We have added few sentences related to the extensive empirical findings in the discussion section which we had highlighted and also the various methodologies and key findings of these articles and robustness of these theories in other different contexts.*

**Comments:** **Critique and Controversies: Discuss any controversies or debates in the literature regarding the relationships between the constructs and entrepreneurial intention. For instance, you briefly mentioned conflicting findings regarding the influence of need for achievement (NACH) on entrepreneurial intention. Expanding on these debates and discussing possible explanations for conflicting results can add depth to your literature review.**

**Responses:** *We have considered your valuable suggestions and incorporated the necessary changes into the in the revised manuscript. We have added few critiques and debates regarding the various constructs in the review of literature section.*

**Comments:Cultural Considerations: Given that your study focuses on engineering students in India, it's important to discuss how cultural factors may shape attitudes, norms, and behaviors related to entrepreneurship in the Indian context. Explore literature that examines cultural dimensions (e.g., collectivism, uncertainty avoidance) and their impact on entrepreneurial intention formation.**

**Responses:** *We have considered your valuable suggestions and incorporated the necessary changes into the in the revised manuscript. We have added the few sentences regarding the cultural factors effect on entrepreneurial intentions in various cross-cultural contexts. We have added few sentences in review of literature and discussion section which we have highlighted.*

**Comments:Please add theoretical implications.**

**Responses:** *We have considered your valuable suggestions and incorporated the necessary changes into the in the revised manuscript. We have added the theoretical implications in the discussion section and also have provided various theoretical implications of these research to the existing theory, entrepreneurship education and the academia.*

**Comments: Lastly, the content is grammatically correct overall. However, few minor issues that could be improved for clarity and coherence. Therefore, proofreading is suggested.**

**Responses:** *We have considered your valuable suggestions and incorporated the necessary changes into the in the revised manuscript. We had done a in depth proof reading of the article and improved the grammatical issues and provided clarity on the paper. It took few times for us in uploading the paper.*

# Reviewer report: Reviewer response for version 1

**DOI:** 10.5256/f1000research.154059.r233045

**Published:** 2023-12-30

**Authors:** Pradeep Kumar Mohanty

**Affiliations:** 1 School of Management, Bennett University, Greater Noida, Uttar pradesh, India

**Dear Editor,**

 The article presents a comprehensive and well-structured study on a relevant topic in entrepreneurship research, with clear implications for policy and education in emerging economies.

**Presentation and Literature Citation:**

- The work is clearly presented, with a structured approach to discussing research objectives, methodology, and findings.
- It cites current literature, particularly emphasizing TPB and its applicability in entrepreneurial studies.

**Study Design and Soundness:**

- The study design, using a structured questionnaire and SEM analysis, is appropriate for the research objectives.
- The work is technically sound, with a robust analytical approach.

**Methods and Analysis Details:**

- The article provides sufficient methodological details, including data collection and analysis methods, allowing for potential replication.

**Statistical Analysis:**

- The statistical analysis, primarily SEM, is appropriate and well-interpreted.
- The authors discuss the significance of various constructs like subjective norms and entrepreneurial motivation in predicting entrepreneurial intention and behaviour.

**Source Data Availability:**

- The article indicates that the underlying data are available, which supports reproducibility.

**Support for Conclusions:**

- The conclusions drawn are adequately supported by the results, aligning with the established theoretical frameworks and the data analysis presented.

**Feedback: **

- The integration of TPB with personality traits and the focus on the intention-behaviour gap is well-justified.
- The methodology and statistical analysis are robust and clearly articulated.
- Addressing broader and more diverse contexts in future research would strengthen the external validity of the findings.
- Overall, the manuscript is commendable.

Is the work clearly and accurately presented and does it cite the current literature?

Yes

If applicable, is the statistical analysis and its interpretation appropriate?

Yes

Are all the source data underlying the results available to ensure full reproducibility?

Yes

Is the study design appropriate and is the work technically sound?

Yes

Are the conclusions drawn adequately supported by the results?

Yes

Are sufficient details of methods and analysis provided to allow replication by others?

Yes

Reviewer Expertise:

Customer Satisfaction & Loyalty, Purchase Intention, Behavior Buyer-Seller relationship, CSR, Consumer behaviour

I confirm that I have read this submission and believe that I have an appropriate level of expertise to confirm that it is of an acceptable scientific standard.
